# Roles of microRNA in aging and dietary restriction-induced longevity

**DOI:** 10.3389/fgene.2026.1832187

**Published:** 2026-07-28

**Authors:** Jialin Fan, Guimei Zhang, Shuo Sun, Xiaowen Zhang, Yaping Li, Meng Liu, Peilin Xu, Mark Youssef, Zara Khanzada, Yifei Zhou, Yunpeng Xu

**Affiliations:** 1 School of Arts and Sciences, Chemistry and Chemical Biology Cancer Institute of New Jersey (CINJ), Director’s Office Cancer Institute of New Jersey (CINJ), Rutgers University, New Brunswick, NJ, United States; 2 Department of Neurology and Neuroscience Center, The First Hospital of Jilin University, Jilin University, Changchun, China; 3 Department of Urology, The Affiliated Hospital of Changchun University of Chinese Medicine, Changchun University of Chinese Medicine, Changchun, China; 4 School of Pharmacy, Shenyang Pharmaceutical University, Shenyang, Liaoning, China; 5 Princeton Neuroscience Institute, Princeton University, Princeton, NJ, United States; 6 Department of Anatomy and Bakar Aging Research Institute, University of California, San Francisco, CA, United States; 7 Department of Molecular Biology and Biochemistry, Rutgers University, Piscataway, NJ, United States; 8 Center for Genomic Medicine and Diabetes Unit, Endocrine Division, Department of Medicine, Massachusetts General Hospital and Harvard Medical School, Boston, MA, United States

**Keywords:** aging, dietary restriction, longevity, microRNAs, muscle aging

## Abstract

Aging is a multifactorial biological process driven by the progressive decline of cellular and tissue homeostasis. Increasing evidence has identified microRNAs (miRNAs) as key epigenetic regulators that fine-tune gene expression networks involved in aging and lifespan determination. Across model organisms—including *Caenorhabditis elegans, Drosophila melanogaster,* and mammals—numerous miRNAs exhibit age-dependent expression patterns and modulate longevity by targeting conserved signaling pathways such as insulin/IGF-1 signaling, mTOR, autophagy, mitochondrial homeostasis, and inflammatory responses. These small non-coding RNAs function as network regulators that coordinate multiple biological processes underlying organismal aging. In addition to regulating intrinsic aging pathways, miRNAs have emerged as important mediators of environmental longevity interventions. Moreover, dietary restriction (DR), one of the most robust and evolutionarily conserved lifespan-extending interventions, reshapes miRNA expression programs that control metabolic adaptation, stress resistance, and nutrient-sensing pathways. Furthermore, accumulating evidence indicates that circulating miRNAs detected in body fluids—including blood, serum, and urine—may serve as minimally invasive biomarkers for biological aging and age-related diseases. In this review, we summarize current insights into the roles of miRNAs in lifespan regulation across model organisms, discuss their involvement in DR-mediated longevity, and highlight their emerging applications as diagnostic biomarkers and potential therapeutic targets. Finally, we discuss future directions emphasizing integrative multi-miRNA regulatory networks and miRNA-based aging clocks, which may provide new opportunities for understanding aging mechanisms and developing strategies to promote healthy longevity.

## 1 Introduction

Aging is a complex and multifactorial biological process characterized by the decline of physiological functions and increased susceptibility to chronic diseases, including neurodegenerative disorders, cardiovascular disease, metabolic dysfunction, and cancer (Lopez-Otin et al., 2023). Over the past 2 decades, accumulating evidence has demonstrated that epigenetic regulation plays a critical role in aging and longevity control. Among these epigenetic regulators, microRNAs (miRNAs) have emerged as key post-transcriptional modulators that fine-tune gene expression networks involved in cellular homeostasis, stress resistance, metabolism, and tissue integrity (de Lucia et al., 2017; Gerasymchuk et al., 2020; Danka Mohammed et al., 2017).

MicroRNAs are small non-coding RNAs approximately 20–24 nucleotides in length that regulate gene expression primarily by binding to complementary sequences in the 3′untranslated regions (3′UTRs) of target mRNAs, resulting in translational repression or mRNA degradation. As each miRNA can regulate multiple targets and each gene can be regulated by multiple miRNAs, these molecules form complex regulatory networks that influence diverse biological processes (Bartel, 2004).

**FIGURE 1:**
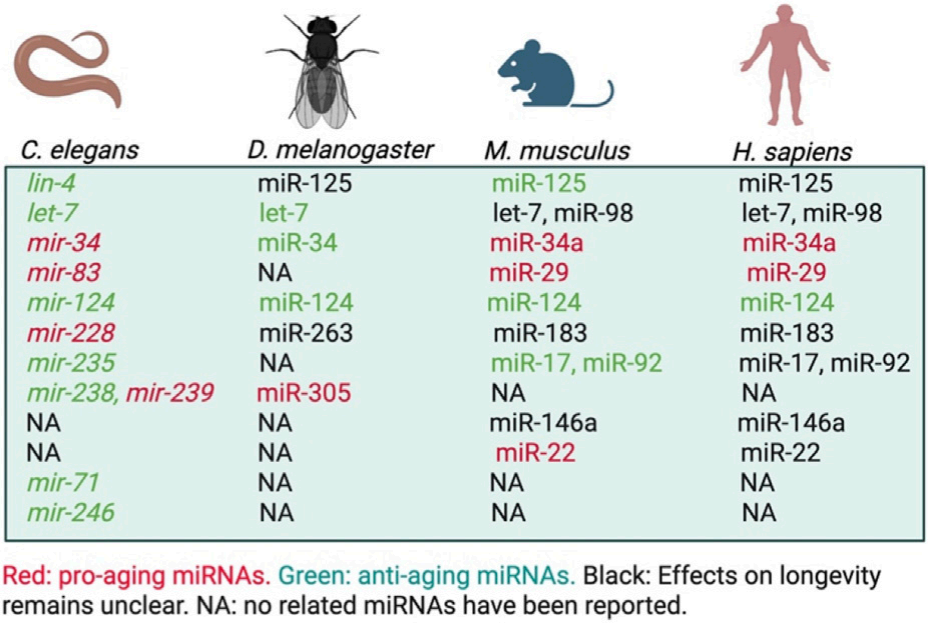
Representative microRNAs regulating lifespan in different model organisms. The diagram summarizes key miRNAs identified in *C. elegans*, *Drosophila*, mice, and humans and their associated signaling pathways. Arrows indicate activation, while blunt lines represent inhibition. Different colors denote miRNAs derived from different species. Abbreviation: NA, not available.

**FIGURE 2:**
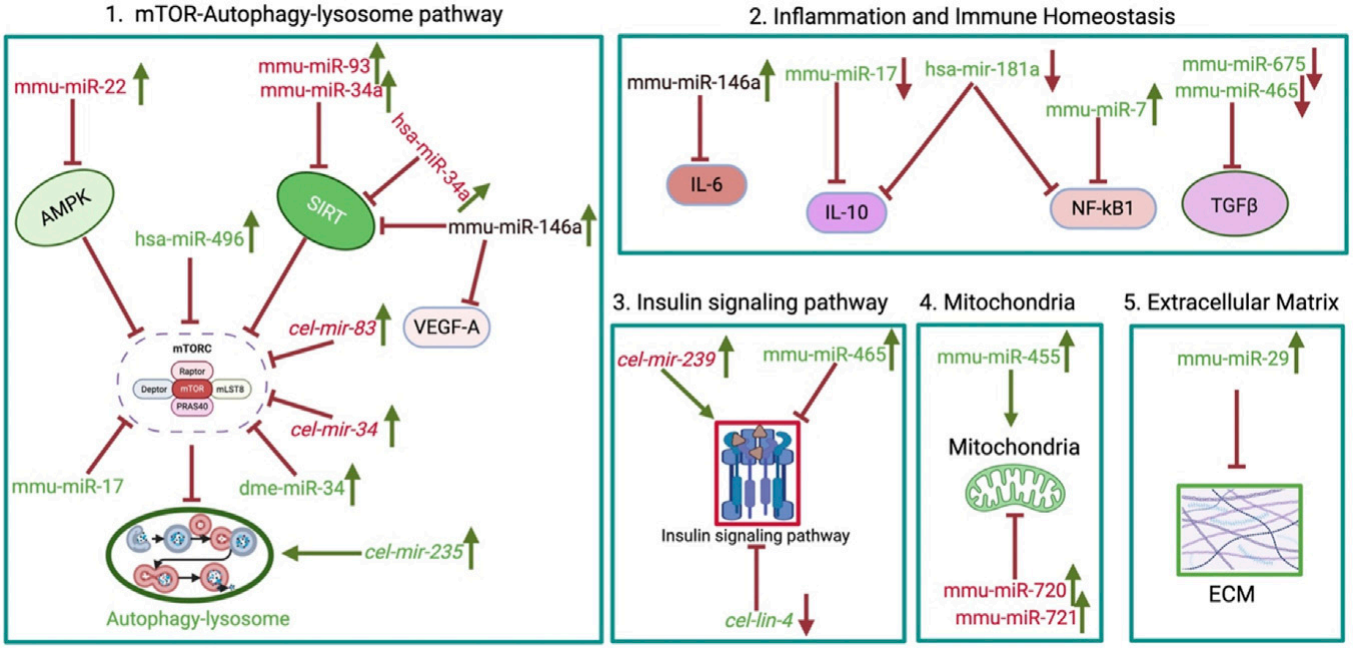
Mechanisms by which microRNAs regulate aging-related signaling pathways. This schematic diagram summarizes major molecular mechanisms through which microRNAs regulate aging-associated biological processes across different species. The figure highlights several key signaling pathways and cellular processes that are modulated by aging-related microRNAs. (1) mTOR–autophagy–lysosome pathway. miRNAs regulate autophagy and lysosomal degradation through modulation of the mTOR signaling network. miRNAs such as mmu-miR-22, mmu-miR-93, mmu-miR-34a, *cel-mir-34, cel-mir-83,* and dme-miR-34 influence upstream regulators including AMPK, SIRT1, and VEGF-A, thereby affecting mTOR activity and autophagy-lysosome function. (2) Inflammation and immune homeostasis. Several miRNAs regulate inflammatory signaling and immune responses during aging. miRNAs including mmu-miR-146a, mmu-miR-17, hsa-miR-181a, mmu-miR-7, mmu-miR-465, and mmu-miR-675 modulate cytokine and immune regulatory pathways involving IL-6, IL-10, NF-κB1, and TGF-β, thereby influencing age-associated inflammation. (3) Insulin signaling pathway. miRNAs regulate metabolic homeostasis and lifespan through the insulin/IGF-1 signaling pathway. For example, *cel-mir-239* and mmu-miR-465 influence components of insulin signaling, while cel-lin-4 has been reported to negatively regulate this pathway. (4) Mitochondrial function. miRNAs also regulate mitochondrial homeostasis during aging. mmu-miR-455, mmu-miR-720, and mmu-miR-721 have been implicated in the control of mitochondrial activity and function. (5) Extracellular matrix (ECM). Certain microRNAs influence aging-related extracellular matrix remodeling. For instance, mmu-miR-29 regulates ECM components and contributes to age-associated tissue structural changes. Together, these miRNA-mediated regulatory networks illustrate how microRNAs coordinate multiple conserved pathways to influence aging and lifespan. Green arrows indicate upregulation, whereas red arrows indicate downregulation of microRNA expression during aging. T-shaped lines represent inhibitory interactions, while arrows represent activation or positive regulation. This figure summarizes representative and well-studied aging-associated microRNAs, including the *miR-34*, *miR-29*, *miR-17*, *miR-146*, and *let-7* families, which have been reported to regulate conserved signaling pathways involved in aging and longevity.

Extensive studies in model organisms—including *C. elegans (Caenorhabditis elegans)*, *D. melanogaster (Drosophila melanogaster)*, and mammals—have revealed that numerous miRNAs exhibit age-dependent expression changes and directly modulate lifespan. These miRNAs influence several conserved longevity pathways, including insulin/IGF-1 signaling, mTOR signaling, autophagy, mitochondrial homeostasis, and inflammatory responses (Ghafouri-Fard et al., 2021; Kinser and Pincus, 2020; Pan et al., 2017; Fan et al., 2019; Fan et al., 2022; Zhao et al., 2024; Fan et al., 2025; Fan et al., 2026). These pathways form an interconnected regulatory system that ultimately determines cellular and organismal aging. The overall regulatory framework through which miRNAs influence these longevity pathways is illustrated in (Figures 1, 2)**.** MiRNAs can function as either pro- or anti-aging factors, modulated by their specific molecular targets and tissue-dependent roles.

In addition to regulating intrinsic aging pathways, miRNAs are also involved in mediating environmental interventions that extend lifespan. Among these interventions, dietary restriction (DR)—defined as reduced nutrient intake without malnutrition—is one of the most robust and evolutionarily conserved strategies known to extend lifespan in organisms ranging from yeast to mammals. Emerging studies have revealed that DR induces dynamic changes in miRNA expression, suggesting that miRNAs may serve as molecular mediators linking environmental signals to longevity pathways (Fan et al., 2025; Makwana et al., 2017; Ozorhan et al., 2022; Yamada et al., 2020; Zhang et al., 2019; Shastri et al., 2020; Xu et al., 2019; Hu et al., 2024; Fan and Xu, 2026).

Furthermore, miRNAs are increasingly recognized as promising biomarkers for aging and age-related diseases. Circulating miRNAs have been detected in blood, serum, urine, and extracellular vesicles, and their expression patterns correlate with chronological age, biological aging markers, and disease risk (Gerasymchuk et al., 2020; Huan et al., 2018; Meng et al., 2015; Luo et al., 2013; Zhang et al., 2015; Swarbrick et al., 2019; Havelka et al., 2025). Because these molecules are stable and detectable through minimally invasive sampling, they represent attractive candidates for clinical diagnostics and therapeutic interventions. The major aging-related miRNAs identified across model organisms and human studies are summarized in (Tables 1–3).

**TABLE 1:** MicroRNAs function in aging across model organisms.

miRNA	Species	Expression*	Tissue	Target	Role
*lin-4* [de Lencastre et al., 2010]	*C. elegans*	↓	Whole animal/neurons	Insulin Signaling pathway (IIS)	Anti-aging
*let-7 [*(de Lencastre et al., 2010)*;* (Ibanez-Ventoso et al., 2006)*]*	*C. elegans*	↓	Whole animal	Developmental timing pathway	Anti-aging
*mir-34 [*(de Lencastre et al., 2010)*;* (Yang et al., 2013)*]*	*C. elegans*	↑	Neurons	atg-9/autophagy	Pro-aging
*mir-71 [*(de Lencastre et al., 2010)*;* (Pincus et al., 2011)*]*	*C. elegans*	↑	Neurons	DAF-16 signaling	Anti-aging
*mir-83 [*(Zhou et al., 2019)*]*	*C. elegans*	↑	Intestine/secreted	Autophagy pathwayy	Pro-aging
*mir-124 [*(Wang et al., 2015)*]*	*C. elegans*	↑	Nervous system	TF-6/ER stress pathway	Anti-aging
*mir-229 [*(Matai et al., 2023)*]*	*C. elegans*	↓	Whole animal	SKN-1/NRF signaling	Anti-aging
*mir-238 [*(de Lencastre et al., 2010)*;* (Chipman et al., 2023)*]*	*C. elegans*	↑	Whole animal	Longevity-associated signaling	Anti-aging
*mir-239 [*(de Lencastre et al., 2010)*;* (Pincus et al., 2011)*]*	*C. elegans*	↑	Whole animal	IIS pathway	Pro-aging
*mir-246 [*(de Lencastre et al., 2010)*;* (Pincus et al., 2011)*]*	*C. elegans*	↑	Whole animal	Stress resistance pathway	Anti-aging
let-7 [(de Lencastre et al., 2010); (Chawla et al., 2016)]	*D. melanogaster*	↑	Brain/neurons	Chinmo	Anti-aging
miR-34 [(Liu et al., 2012a)]	*D. melanogaster*	↑	Brain	Neuroprotection pathway	Anti-aging
miR-305 [(Ueda et al., 2018)]	*D. melanogaster*	↓	Intestine	Metabolic signaling	Pro-aging
miR-7 [(Wang et al., 2024); (Zhang et al., 2021)]	*M. musculus*	↑	Liver Kupffer cells; brain	KLF4	Anti-aging
miR-17 [(Zhang et al., 2020); (Du et al., 2014); (Grandke et al., 2026)]	*M. musculus*	↓	Adipose tissue, macrophages	MKP7, IL-10 signaling	Anti-aging
miR-22 [(Jazbutyte et al., 2013); (Smith-Vikos and Slack, 2012)]	*M. musculus*	↑	Cardiac fibroblasts	Senescence-associated signaling	Pro-aging
miR-29 [(Takeda and Tanabe, 2016); (Swahari et al., 2024)]	*M. musculus*	↑	Skeletal muscle; brain	ECM signaling	Pro- or anti-aging
miR-34a (Fulzele et al., 2019; Yang et al., 2013; Li et al., 2011b)	*M. musculus*	↑	Skeletal muscle EVs; heart; brain	SIRT1	Pro-aging
miR-93 [(Li et al., 2011a)]	*M. musculus*	↑	Liver	SIRT1	Pro-aging
mir-124 [(Liu et al., 2022)]	*M. musculus*	↓	Brain	RyR3	Anti-aging
miR-146a (Hao et al., 2016; Gong et al., 2022)	*M. musculus*	↑	RPE cells	IL-6, VEGF-A, NAMPT	Pro- or anti-aging
miR-434 [Pardo et al., 2017]	*M. musculus*	↓	Skeletal muscle	eIF5A1	Anti-aging
miR-455 [(Jung et al., 2017)]	*M. musculus*	↓	Brain	Mitochondrial homeostasis	Anti-aging
miR-465 [(Elias et al., 2019)]	*M. musculus*	↑	Liver	Growth hormone signaling	Pro-aging
miR-468 [(Li et al., 2011b)]	*M. musculus*	↑	Brain	Oxidative phosphorylation	Pro-aging
miR-496 [(Rubie et al., 2016)]	*M. musculus*	↑	PBMCs	mTOR pathway	Anti-aging
miR-542 [(Qian et al., 2015)]	*M. musculus*	↓	VSMCs	Vascular remodeling pathway	Anti-aging
miR-669 b (Mula et al., 2023)	*M. musculus*	↑	Neuro2a APP cells	CHEK2	Anti-aging
miR-669c (Kolosowska et al., 2020)	*M. musculus*	↑	Microglia/macrophages	MyD88	Anti-aging
miR-675 [(Han et al., 2019)]	*M. musculus*	↓	Vascular endothelium	TGF-β/vascular aging pathways	Anti-aging
miR-720 [(Li et al., 2011b)]	*M. musculus*	↑	Brain	Mitochondrial pathway	Pro-aging
miR-721 [(Li et al., 2011b)]	*M. musculus*	↑	Brain	Mitochondrial pathway	Pro-aging

Table 1 This table summarizes representative microRNAs that have been experimentally shown to regulate lifespan in major model organisms, including *C. elegans, D. melanogaster*, mice, and humans. The table lists each miRNA, the species in which it was studied, the direction of expression change during aging, and its reported role in lifespan regulation (pro-aging or anti-aging). Expression changes indicate whether the miRNA level increases (↑) or decreases (↓) during aging or in aged tissues. The functional classification reflects experimental evidence showing whether the miRNA promotes lifespan extension (anti-aging) or accelerates aging processes (pro-aging).

Abbreviations: Expression*: expression during aging or aged tissues. ATG9, autophagy-related protein 9; BTB-ZF, Broad-complex, Tramtrack and Bric zinc finger transcription factor; CHEK2, checkpoint kinase 2; ECM, extracellular matrix; EVs, extracellular vesicles; FOXO, forkhead box O transcription factor; GH, growth hormone; HASMCs, human aortic smooth muscle cells; IIS, insulin/insulin-like growth factor-1, signaling; IL-6, interleukin-6; IL-10, interleukin-10; KLF4, Kruppel-like factor 4; MKP7, mitogen-activated protein kinase phosphatase 7; mTOR, mechanistic target of rapamycin; MyD88, myeloid differentiation primary response protein 88; NAMPT, nicotinamide phosphoribosyltransferase; NRF2, nuclear factor erythroid 2-related factor 2; PBMCs, peripheral blood mononuclear cells; RPE, retinal pigment epithelium; RyR3, ryanodine receptor 3; SIRT1, sirtuin 1; TGF-β, transforming growth factor-beta; UPRmt, mitochondrial unfolded protein response; VEGF-A, vascular endothelial growth factor A; VSMCs, vascular smooth muscle cells.

**TABLE 2:** Aging-related and neurodegenerative disease-associated microRNAs in human.

miRNA	Expression*	Sample source	Association	Tissue
miR-7 [(Ramesh and Govindaraju, 2025)]	↑	Brain tissue	Aging	Brain
miR-34a (Li et al., 2011c)	↑	Brain tissue	Aging	Brain
miR-124 [Liu et al., 2022]	↓	Brain tissue	Aging	Brain
let-7e, miR-98, 1268 [(Drummond et al., 2011)]	↑	Skeletal muscle biopsy	Aging	Muscle
miR-22, 223, 378 [Drummond et al., 2011]	↓	Skeletal muscle biopsy	Aging	Muscle
miR-22 [Huan et al., 2018]	↑	Peripheral blood	Aging	Blood
miR-92a, 222, 375 [Zhang et al., 2015]	↑	Serum	Aging	Blood
miR-29b, 106b, 130b, 142, 340 [Zhang et al., 2015]	↓	Serum	Aging	Blood
miR-99b, 496, 505 [Huan et al., 2018]	↓	Peripheral blood	Aging	Blood
miR-151a, 181a, 1248 [(Noren Hooten et al., 2013)]	↓	Serum	Aging	Blood
miR-31, 34a, 146a, 155 [Havelka et al., 2025]	↑	Urine	Aging biomarker	Urine
miR-9 [Delay et al., 2012]	↑/↓ in AD patients	Brain tissue	Alzheimer’s disease	Brain
miR-29, 107 [Delay et al., 2012]	↓ in AD patients	Brain tissue	Alzheimer’s disease	Brain
miR-34, 545 [(Cosin-Tomas et al., 2017)]	↓ in AD patients	Plasma	Alzheimer’s disease	Blood
miR-106 b (Madadi et al., 2022; Liu et al., 2016)	↓ in AD patients	Brain, blood	Alzheimer’s disease	Brain, blood
miR-146a, 181a (Ansari et al., 2019)	↓ in AD patients	Brain tissue	Alzheimer’s disease	Brain

Table 2 This table summarizes microRNAs whose expression levels have been reported to change in human aging or aging-associated diseases, particularly neurodegenerative disorders such as Alzheimer’s disease (AD). For each miRNA, the table lists its direction of expression change and the tissue or biological sample in which the change was detected. Changes marked as ↑ indicate increased expression during aging or disease, whereas ↓indicates decreased expression. In some cases, miRNAs may show variable expression depending on disease stage, tissue type, or experimental conditions.

Abbreviations: Expression*: expression during aging or aged tissues. AD, Alzheimer’s disease; PBMCs, peripheral blood mononuclear cells.

**TABLE 3:** MicroRNAs involved in dietary restriction-mediated lifespan regulation.

miRNA	Species	Target	DR paradigm	Expression*	Function
*mir-80* [Vora et al., 2013]	*C. elegans*	Unknown	Genetic DR mimic	↓	Activates DR-like longevity program
*mir-235* [Xu et al., 2019; Hu et al., 2024]	*C. elegans*	*cwn-1/WNT4*	DR	↑	Wnt-mediated longevity
*mir-71* [Smith-Vikos et al., 2014]	*C. elegans*	*pha-4/FOXA*	DR	↑	Stress resistance
*mir-228* [Smith-Vikos et al., 2014]	*C. elegans*	*pha-4/FOXA, skn-1/Nrf*	DR	↑	Oxidative stress response
*mir-229* [Matai et al., 2023]	*C. elegans*	*odd-2/OSR, skn-1/Nrf*	DR	↑	Nutrient sensing and longevity
let-7, miR-184/125 [(Gendron and Pletcher, 2017)]	*D. melanogaster*	*chinmo/ZBTB*	DR	↑	Metabolic adaptation
miR-16 [Yamada et al., 2020]	*M. musculus*	*IL-1β, IL-6, TNF-α*	CR	↑	Anti-inflammatory response
miR-29/30 [Shastri et al., 2020]	*M. musculus*	*IGF-1/IGF-1R*	CR	↑	Metabolic remodeling
miR-122 [Zhang et al., 2019]	*M. musculus*	UPR^mt^	CR	↑	Mitochondrial proteostasis
miR-125a (Makwana et al., 2017)	*M. musculus*	*STAT3, CASP2, STARD13*	CR	↑	Stress resistance
miR-184 [Ozorhan et al., 2022]	*M. musculus*	Unknown	CR	↓	Brain aging adaptation
miR-126 [(Donghui et al., 2019)]	*H. sapiens*	Nrf	Diet and exercise intervention	↑	Endothelial protection

Table 3 This table summarizes microRNAs whose expression or function has been reported to change under dietary restriction conditions across different species. For each miRNA, the corresponding organism, target genes or pathways, and major biological functions are listed. These miRNAs regulate key processes involved in DR-induced longevity, including metabolic remodeling, stress resistance, inflammatory regulation, and nutrient-sensing pathways such as insulin/IGF-1 signaling and FOXO-dependent transcription.

Symbols: ↑ increased expression under DR; ↓ decreased expression under DR; NC, not clearly defined or not experimentally validated.

Abbreviations: Expression*: expression change in DR, treatment; CR, calorie restriction; DR, dietary restriction; IIS, insulin/insulin-like growth factor-1, signaling; NRF2, nuclear factor erythroid 2-related factor 2; PHA-4, pharynx defective 4; SKN-1, skinhead-1; STAT3, signal transducer and activator of transcription 3; UPRmt, mitochondrial unfolded protein response; WNT, Wingless/Integrated signaling pathway.

In this review, we summarize current knowledge on the roles of microRNAs in aging and lifespan regulation across model organisms, discuss their involvement in dietary restriction-mediated longevity and evaluate their potential applications as biomarkers and therapeutic targets in human aging.

## 2 MicroRNA biogenesis and regulatory principles

MicroRNAs are generated through a multistep biogenesis pathway that involves both nuclear and cytoplasmic processing. Most miRNAs are transcribed by RNA polymerase II as long primary transcripts known as pri-miRNAs. These transcripts contain one or more stem-loop structures that are recognized and processed in the nucleus by the microprocessor complex composed of the RNase III enzyme Drosha and its cofactor DGCR8. The Drosha-mediated cleavage produces precursor miRNAs (pre-miRNAs), which are approximately 70 nucleotides in length and possess a characteristic hairpin structure. These pre-miRNAs are subsequently exported from the nucleus to the cytoplasm by Exportin-5. In the cytoplasm, another RNase III enzyme, Dicer, further processes the pre-miRNA into a ∼22-nucleotide double-stranded RNA duplex. One strand of this duplex is incorporated into the RNA-induced silencing complex (RISC), where it guides the complex to complementary sequences within target mRNAs. The degree of sequence complementarity between the miRNA and its target determines the mechanism of repression, which may involve translational inhibition or mRNA degradation (Bartel, 2004). As miRNAs regulate large networks of genes simultaneously, they are particularly well suited to coordinate complex biological processes such as development and aging. In many cases, miRNAs act as fine-tuning regulators that stabilize gene expression programs rather than acting as simple on/off switches. Importantly, the expression and activity of core biogenesis enzymes, particularly Dicer and Drosha, are highly sensitive to chronological aging. Age-related reductions in these components, notably Dicer in adipose tissues, lead to compromised global miRNA maturation and a consequential redistribution of the cellular miRNA profile (Sanz-Ros et al., 2023).

## 3 miRNAs and lifespan regulation in model organisms

### 3.1 miRNAs in *Caenorhabditis elegans*

*Caenorhabditis elegans* plays a central role in the discovery of miRNA functions in aging. Early studies revealed that numerous miRNAs exhibit dynamic expression changes during the lifespan of the worm. Functional analysis demonstrated that some of these miRNAs can either extend or shorten lifespan (Table 1). One of the best-studied longevity-associated miRNAs is miR-71. This miRNA increases with age and promotes lifespan extension by regulating stress response pathways and neuronal signaling. Loss of miR-71 results in reduced lifespan and impaired stress resistance. Interestingly, neuronal expression of miR-71 can influence systemic aging, highlighting the importance of neuroendocrine regulation in longevity (de Lencastre et al., 2010). Another important miRNA is miR-239, which functions as a pro-aging factor. Overexpression of miR-239 shortens lifespan, whereas loss of this miRNA extends lifespan. These opposing effects illustrate how different miRNAs can modulate aging through distinct molecular targets (de Lencastre et al., 2010). Additional miRNAs involved in nematode aging include miR-34, miR-238, miR-246, and miR-83. These miRNAs regulate pathways associated with autophagy, stress resistance, and metabolic homeostasis (Figures 1, 2). Notably, several miRNAs regulate lifespan in *C. elegans* by interacting with classical aging pathways. For example, some miRNAs influence insulin signaling by modulating DAF-16/FOXO activity, thereby altering lifespan and stress resistance. In addition, certain miRNAs are involved in mTOR and AMPK signaling networks, highlighting their important roles in metabolic regulation and energy sensing.

### 3.2 miRNAs in *Drosophila melanogaster*

Research in fruit flies has provided further insights into the role of miRNAs in aging and neurodegeneration. One of the most prominent examples is miR-34, which increases during aging and protects against age-related neurodegeneration. Flies lacking miR-34 exhibit shortened lifespan and increased neuronal degeneration (Table 1) (Liu et al., 2012a).

The miR-125/let-7 regulatory axis is another critical pathway influencing lifespan in flies. These miRNAs regulate the transcription factor Chinmo, which plays important roles in neuronal differentiation and metabolic regulation. Changes in this pathway affect both metabolism and longevity (Chawla et al., 2016). Other miRNAs identified in fly aging include miR-305, which promotes aging by influencing metabolic gene expression in muscle and intestinal tissues (Ueda et al., 2018).

### 3.3 miRNAs in mammalian aging

In mammals, miRNAs regulate aging processes in multiple tissues, including brain, muscle, liver, and immune cells. Many miRNAs show age-dependent expression changes and influence key pathways involved in cellular senescence, inflammation, and metabolic regulation. One of the most widely studied aging-associated miRNAs is miR-34a (Fulzele et al., 2019; Raucci et al., 2021). This miRNA increases with age in several tissues and contributes to cellular senescence by targeting genes involved in mitochondrial function and DNA repair. Another important longevity-associated miRNA is miR-17, which has been shown to extend lifespan in mice by suppressing senescence signaling pathways. MiR-17 also plays roles in immune regulation and tissue homeostasis (Zhang et al., 2020; Du et al., 2014). In skeletal muscle, miRNAs such as miR-434 and miR-455 regulate apoptosis and muscle aging, whereas miR-22 contributes to cardiac aging by promoting fibroblast activation (Kumar et al., 2021; Pardo et al., 2017; Jazbutyte et al., 2013).

Overall, studies across model organisms demonstrate that miRNAs are conserved regulators of lifespan and aging-related processes. Although individual miRNAs may differ between species, many of them converge on common biological pathways, including stress response, metabolic regulation, autophagy, and neuronal signaling (Figures 1, 2). These small non-coding RNAs function as fine-tuning modulators that coordinate gene expression networks underlying aging. Importantly, several miRNAs identified in invertebrates, such as miR-34 and the miR-125/let-7 family, show conserved roles in mammalian aging, suggesting evolutionary conservation of miRNA-mediated longevity control. Therefore, model organisms provide valuable systems for uncovering fundamental miRNA regulatory mechanisms that may ultimately inform therapeutic strategies targeting human aging and age-related diseases.

This context-dependent dichotomy is heavily shaped by both evolutionary species-specific divergence and the paradigm of antagonistic pleiotropy. For instance, while miR-34 function is conserved in promoting p53-mediated apoptosis and cellular senescence across mammals, its impact on longevity varies dramatically between model organisms; its ablation shortens lifespan in *C. elegans* but extends it in *Drosophila* by mitigating neurodegeneration and proteotoxicity (Liu et al., 2012a; Li et al., 2011a; Yang et al., 2013). This demonstrates that a single miRNA can exert pro- or anti-aging phenotypes depending not only on the tissue-specific microenvironment but also on the distinct genetic backgrounds and downstream target networks inherent to each species.

Importantly, the strength of evidence supporting aging-associated miRNAs varies considerably among species. In model organisms such as *C. elegans*, *Drosophila*, and mice, many miRNAs have been functionally validated using genetic deletion, overexpression, or tissue-specific manipulation approaches, thereby demonstrating causal roles in lifespan regulation. Examples include miR-71, miR-80, miR-228/229, and miR-239 in *C. elegans*, whose perturbation directly alters longevity phenotypes. In contrast, most human aging-associated miRNAs have been identified through observational or profiling studies and therefore currently serve primarily as biomarkers rather than experimentally proven drivers of aging. Distinguishing causal aging regulators from aging-associated biomarkers will be essential for translating miRNA biology into therapeutic interventions.

## 4 MicroRNAs in age-related diseases

Age-related diseases share many molecular mechanisms with the aging process itself, and dysregulation of miRNAs has been implicated in the pathogenesis of numerous age-associated disorders. Neurodegenerative diseases such as Alzheimer’s disease are closely associated with changes in miRNA expression. Several miRNAs involved in neuronal development and synaptic function are altered in AD patients (Table 2) (Swarbrick et al., 2019; Delay et al., 2012). For example, miR-29 and miR-107 are downregulated in the brains of individuals with Alzheimer’s disease and regulate genes involved in amyloid precursor protein processing. MiR-106 b has also been implicated in AD pathology by influencing tau phosphorylation. Another important miRNA is miR-146a, which regulates inflammatory signaling pathways in the brain. Dysregulation of this miRNA may contribute to chronic neuroinflammation during aging (Table 2).

## 5 MicroRNAs in dietary restriction-induced longevity

Dietary restriction (DR), defined as reduced nutrient intake without malnutrition, is one of the most robust and evolutionarily conserved interventions known to extend lifespan. Dietary restriction (DR) represents a collection of nutritional interventions rather than a single experimental paradigm. The implementation of DR varies substantially among species. In *C. elegans*, DR is commonly achieved by reducing bacterial food concentration or altering feeding schedules, whereas in *Drosophila*, both food quantity and dietary composition can be manipulated. In mammals, researchers use a broader range of dietary interventions, including caloric restriction, protein restriction, amino acid restriction, intermittent fasting, and time-restricted feeding. These distinct dietary regimens can induce different microRNA expression profiles and activate partially overlapping nutrient-sensing pathways, including insulin/IGF-1 signaling, mTOR, AMPK, and FGF21-mediated metabolic adaptation (Elliott et al., 2025). Therefore, the effects of DR-associated miRNAs should be interpreted within the specific dietary context in which they are studied. Increasing evidence indicates that DR reshapes microRNA expression patterns and that specific miRNAs play essential roles in mediating the longevity benefits of nutrient limitation. These miRNAs function by modulating key transcription factors, metabolic regulators, and stress-response pathways that are central to DR signaling (Fan et al., 2025; Xu et al., 2019; Hu et al., 2024; Fan and Xu, 2026).

In *C. elegans*, several miRNAs have been identified as critical regulators of DR-induced longevity. Notably, deletion of miR-80 mimics dietary restriction and significantly extends lifespan by regulating metabolic adaptation pathways (Vora et al., 2013). Additional miRNAs such as miR-71, miR-228, and miR-229 contribute to DR-associated lifespan extension by modulating transcription factors including PHA-4 and SKN-1, which control oxidative stress responses, detoxification pathways, and metabolic homeostasis (Smith-Vikos et al., 2014). These findings suggest that miRNAs act as upstream regulators linking nutrient sensing to transcriptional programs that promote stress resistance and survival under nutrient-limited conditions.

In *D. melanogaster*, DR-mediated longevity has also been linked to miRNA-dependent regulatory networks. The miR-125–Chinmo axis plays an important role in mediating lifespan extension under DR conditions by controlling the expression of metabolic genes involved in lipid metabolism and energy balance (Pandey et al., 2021). This pathway illustrates how miRNAs can coordinate endocrine and metabolic responses to nutrient availability.

In mammals, dietary restriction similarly induces widespread changes in miRNA expression across multiple tissues. For example, miR-16 is upregulated in calorie-restricted mice and contributes to reduced inflammatory signaling by suppressing the expression of pro-inflammatory cytokines. Other miRNAs such as miR-29 and miR-30 regulate components of the insulin/IGF-1 signaling pathway, which is a central regulator of metabolism and lifespan. Through these targets, DR-responsive miRNAs influence metabolic remodeling, mitochondrial function, and cellular stress resistance (Shastri et al., 2020).

To understand the organismal impact of dietary restriction (DR), it is essential to decouple cell-autonomous, tissue-specific miRNA responses from systemic ‘hormetic’ signaling networks. Emerging paradigms reveal that local energetic stress—particularly within energy-regulating tissues such as the gut and adipose tissue—triggers the secretion of specific miRNAs encapsulated within extracellular vesicles (EVs) (Ferrante et al., 2015). These EV-miRNAs enter the systemic circulation to function as endocrine-like mediators of inter-organ communication (Zhao et al., 2021). Through this vesicle-mediated signaling, local nutrient-sensing events can be transmitted to distant tissues, coordinating systemic metabolic adaptation, and ultimately contributing to longevity.

Although most currently characterized longevity-associated miRNAs have been identified in classical dietary restriction (DR) and calorie restriction (CR) paradigms, emerging evidence suggests that other dietary interventions may also engage miRNA-mediated regulatory mechanisms (Fan et al., 2025; Fan and Xu, 2026). For example, protein restriction (PR) and amino acid restriction (AAR), particularly methionine restriction, modulate FGF21, mTOR, GCN2, and ATF4 signaling pathways that are closely linked to aging and longevity. Several miRNAs, including miR-133a, miR-206, miR-210, and members of the miR-29 family, have been implicated in nutrient-sensing and metabolic adaptations associated with these interventions (Elliott et al., 2025; Gallinetti et al., 2013; Mirzaei et al., 2014; Giacomello and Toniolo, 2021). However, direct evidence linking these miRNAs to lifespan extension under PR or AAR remains limited.

Similarly, intermittent fasting (IF), time-restricted feeding (TRF), and fasting-mimicking diets (FMDs) induce distinct metabolic and circadian adaptations that may involve miRNA regulation. Studies have reported altered expression of several aging-associated miRNAs, including miR-21, miR-34a, miR-146a, and the liver-enriched miR-122, in response to dietary and metabolic interventions. Circadian-associated miRNAs, such as miR-132 and miR-219, may also contribute to TRF-related metabolic remodeling (Elliott et al., 2025; Gallinetti et al., 2013). Nevertheless, the functional roles of these miRNAs in mediating the longevity benefits of IF, TRF, or FMDs remain largely unexplored. Collectively, these findings highlight an important knowledge gap and support recent proposals that future studies should focus on mechanistic validation of diet-responsive miRNAs rather than expression profiling alone.

A summary of representative DR-associated miRNAs across different model organisms and mammalian systems is provided in (Table 3), including their species distribution, target pathways, and functional roles in metabolic adaptation and lifespan regulation.

## 6 Circulating microRNAs as potential biomarkers for human aging and age-related diseases

Large-scale profiling studies have identified numerous circulating microRNAs (miRNAs) associated with human aging. These miRNAs can be detected in body fluids such as blood, serum, urine, and extracellular vesicles. Several circulating miRNAs exhibit age-dependent expression patterns. For example, miR-22, miR-92a, and miR-222 have been reported to increase with age in human serum, whereas miR-29b, miR-106b, and miR-130 b decrease during the aging process (Huan et al., 2018; Zhang et al., 2015). Because circulating miRNAs are relatively stable and can be obtained through minimally invasive sampling, they have attracted considerable attention as potential biomarkers of biological aging. Beyond their association with chronological aging, circulating miRNAs have also been widely investigated as diagnostic biomarkers for age-related diseases. For instance, microRNAs have been extensively studied as biomarkers in cancer patients. In neurodegenerative diseases such as Alzheimer’s disease (AD), which is strongly associated with aging, specific miRNAs also show disease-related alterations. MiR-101a has been reported to be significantly downregulated in AD patients, suggesting its potential value as a diagnostic biomarker, whereas miR-455–3p is increased in AD patients. Overall, a growing number of studies are investigating microRNA profiles in human tissues and body fluids—including skin, urine, blood, and serum—to identify biomarkers that may help predict biological aging and facilitate the early diagnosis of aging-related diseases (Gerasymchuk et al., 2020; Swarbrick et al., 2019; Havelka et al., 2025).

While circulating miRNAs are promising clinical biomarkers, technical hurdles limit their practical use. The main issue is the lack of a standardized reference control to normalize data (the ‘housekeeping’ miRNA problem) (Salama et al., 2024). Additionally, differences in how blood samples are collected, processed, and extracted make study results inconsistent. Biological variables including sex, ethnicity, medication use, and comorbidities may further confound circulating miRNA profiles. Moreover, the tissue origin of many circulating miRNAs remains difficult to determine, and differences among analytical platforms contribute additional variability across studies. Overcoming these technical and biological sources of variation through standardized protocols and large-scale validation studies will be essential before translating circulating miRNAs into clinical biomarkers.

## 7 MicroRNAs in skeletal muscle aging and sarcopenia

Skeletal muscle aging is characterized by a progressive decline in muscle mass, strength, and regenerative capacity, a condition commonly referred to as sarcopenia. This process is driven by multiple molecular alterations, including impaired satellite cell function, mitochondrial dysfunction, chronic inflammation, and dysregulated protein homeostasis. Increasing evidence suggests that microRNAs (miRNAs) play critical regulatory roles in skeletal muscle aging by fine-tuning gene expression networks involved in muscle maintenance, regeneration, and metabolic homeostasis (Fan et al., 2025).

A distinction should be made between muscle-enriched microRNAs (myomiRs), circulating muscle-derived miRNAs, and extracellular vesicle (EV)-associated miRNAs. MyomiRs, including miR-1, miR-133, miR-206, and miR-486, are preferentially expressed in skeletal and cardiac muscle and primarily regulate local processes such as myogenesis, satellite cell activation, neuromuscular junction (NMJ) maintenance, mitochondrial quality control, and muscle regeneration (Chen et al., 2006; Liu et al., 2007; McCarthy, 2008; Blondal et al., 2013). In contrast, circulating muscle-derived miRNAs and EV-associated miRNAs may function as endocrine-like signaling molecules that mediate inter-organ communication during aging and metabolic adaptation. Emerging evidence suggests that age-associated alterations in these miRNAs contribute to impaired satellite-cell function, mitochondrial dysfunction, chronic inflammation, fibrosis, and anabolic resistance, all of which are major hallmarks of sarcopenia. Furthermore, lifestyle interventions including dietary restriction, fasting, and exercise can partially reverse age-related miRNA dysregulation, suggesting that miRNAs may serve as important mediators linking environmental interventions to the preservation of skeletal muscle function during aging.

Among these regulators, several muscle-enriched miRNAs, have been extensively studied. For example, miR-1, miR-133, and miR-206 are highly expressed in skeletal muscle and are essential for myogenesis and muscle regeneration. MiR-1 promotes myogenic differentiation by targeting histone deacetylase 4 (HDAC4) and other regulators of muscle gene expression, whereas miR-133 primarily stimulates myoblast proliferation (Chen et al., 2006). MiR-206 plays a key role in muscle regeneration by regulating satellite cell activation and neuromuscular junction stability. Age-associated alterations in the expression of these miRNAs have been linked to impaired muscle regeneration and reduced muscle function in aged organisms (Liu et al., 2012b).

Another miRNA implicated in skeletal muscle aging is miR-29, which has been shown to increase during aging in several tissues. Elevated miR-29 levels can promote cellular senescence and contribute to age-related tissue degeneration by targeting genes involved in extracellular matrix regulation and cell survival pathways. In skeletal muscle, increased miR-29 expression has been associated with reduced regenerative capacity and muscle atrophy (Hu et al., 2014).

In addition, miR-486 has emerged as an important regulator of muscle homeostasis through its modulation of the insulin/IGF-1 signaling pathway. MiR-486 can enhance AKT signaling and promote muscle growth and survival by targeting negative regulators of the pathway, such as PTEN and FOXO1. Reduced expression of miR-486 has been reported in aged muscle, which may contribute to impaired anabolic signaling and increased susceptibility to muscle wasting (Alexander et al., 2014).

Mitochondrial dysfunction is another hallmark of skeletal muscle aging, and several miRNAs have been implicated in regulating mitochondrial metabolism and oxidative stress responses in muscle cells. Dysregulation of these miRNAs may disrupt mitochondrial quality control and energy metabolism, thereby accelerating muscle functional decline. Furthermore, chronic low-grade inflammation during aging can alter miRNA expression profiles in skeletal muscle, contributing to the development of sarcopenia.

Interestingly, lifestyle interventions known to promote healthy aging, such as exercise and dietary restriction (DR), have also been shown to modulate the expression of multiple muscle-related miRNAs. These interventions may improve muscle function partly through miRNA-mediated regulation of metabolic pathways, mitochondrial biogenesis, and stress response mechanisms. Therefore, miRNAs represent an important molecular link connecting environmental interventions with the maintenance of skeletal muscle integrity during aging.

Collectively, accumulating evidence indicates that miRNAs play multifaceted roles in skeletal muscle aging by coordinating pathways involved in myogenesis, metabolic regulation, mitochondrial function, and cellular stress responses. A better understanding of miRNA-mediated regulatory networks in skeletal muscle may provide new opportunities for developing therapeutic strategies aimed at preventing or treating sarcopenia and other age-related muscle disorders (Murach and McCarthy, 2017; Jung et al., 2019; Kalan et al., 2025; Liu and Dong, 2025).

## 8 Future perspectives: multi-microRNA signatures for aging prediction and therapeutic applications

Although numerous studies have identified individual microRNAs associated with aging and age-related diseases, increasing evidence suggests that single miRNA markers often lack sufficient sensitivity and specificity for reliable prediction or therapeutic targeting. Aging is a highly complex and multifactorial biological process involving extensive regulatory networks that span multiple signaling pathways, tissues, and cellular processes. Therefore, future research should focus on multi-microRNA signatures and integrated regulatory networks rather than relying on individual miRNAs.

Recent studies indicate that combinations of several miRNAs can provide more robust predictive power for biological aging and disease risk. Because individual miRNAs often regulate multiple targets while a single gene may also be controlled by multiple miRNAs, constructing miRNA–target regulatory networks may significantly improve the accuracy of aging biomarkers. Integrating multiple miRNAs with their predicted or experimentally validated targets can help identify key pathways involved in aging processes such as mitochondrial dysfunction, inflammation, proteostasis, and metabolic regulation.

In addition, the development of multi-miRNA aging clocks based on circulating miRNA profiles represents a promising direction for aging research. Compared with single biomarker approaches, these multi-component models can better capture the complexity of aging-related molecular changes and may allow more precise prediction of biological age, disease susceptibility, and lifespan trajectories. Such models could potentially integrate data from plasma, serum, extracellular vesicles, or tissue-specific miRNAs.

Despite these promising opportunities, several challenges remain for translating miRNA research into clinical applications. These include validating miRNA signatures across large and diverse human cohorts and minimizing off-target effects in miRNA-based therapies. Addressing these challenges will be essential for harnessing the full potential of miRNAs in aging research and precision medicine.

Future studies may integrate multi-omics data with machine learning approaches to construct predictive models of aging based on miRNA signatures. By combining the expression patterns of multiple circulating miRNAs, it may be possible to develop a “miRNA aging clock” for estimating biological age and predicting the risk of age-related diseases.

In summary, the future of miRNA research in aging is likely to shift from single-molecule studies toward integrative multi-miRNA regulatory networks that combine biomarker discovery, mechanistic insights, and therapeutic development. Such approaches may ultimately enable more accurate prediction of biological aging and provide novel strategies for preventing or treating age-related diseases.
